# L-VALINE TREATMENT IMPROVES CELLULAR MITOCHONDRIAL FUNCTION

**DOI:** 10.1093/geroni/igad104.2999

**Published:** 2023-12-21

**Authors:** Shakshi Sharma, Gohar Azhar, Xiaomin Zhang, Jeanne Wei

**Affiliations:** University of Arkansas for Medical Sciences, Little Rock, Arkansas, United States; University of Arkansas for Medical Sciences, Little Rock, Arkansas, United States; University of Arkansas for Medical Sciences, Little Rock, Arkansas, United States; University of Arkansas for Medical Sciences, Little Rock, Arkansas, United States

## Abstract

Branched-chain amino acids (BCAAs) are pivotal for the health of the human body. Leucine, isoleucine, and valine, which make up the BCAAs, are α-amino acids that promote skeletal muscle growth and development. Valine is one of the essential BCAAs required for the synthesis of muscle proteins and the support growth of skeletal muscles. Valine also plays a favorable role in enhancing insulin sensitivity, preserving intestinal health, and optimizing lipid metabolism. Considering the relevance of valine in promoting muscle growth and metabolism, we utilized C2C12 skeletal muscle cell line to investigate the role of valine in regulating mitochondrial functions. C2C12 cells grown on physiological normal glucose media (100mg/dL) were used for the experiments. Cells were treated with a 1.0 mM concentration of valine for 24 h. The effect of valine treatment on gene expression of important mitochondrial-related genes such as PGC-1α, and PGC-1β and mitofusin (MFN1, MFN), and mitochondrial fission 1 (Fis 1) was determined. Further, Oroboros oxygraph-O2K high-resolution respirometer was used to analyze the mitochondrial respiration in the intact C2C12 cells after valine treatment. The results showed increased gene expression of PGC-1α, PGC-1β, and mitochondrial fission and fusin genes after treatment with valine. The basal respiration, leak respiration, as well as maximal capacity of mitochondrial ETC (Electron Transport Chain), were also significantly improved after valine treatment. The findings from this study enhance our understanding of valine as an important nutrient to improve mitochondrial function to drive cellular function and biological processes.

